# Mindful Leader Development: How Leaders Experience the Effects of Mindfulness Training on Leader Capabilities

**DOI:** 10.3389/fpsyg.2019.01081

**Published:** 2019-05-15

**Authors:** Silke Rupprecht, Pia Falke, Niko Kohls, Chris Tamdjidi, Marc Wittmann, Wendy Kersemaekers

**Affiliations:** ^1^Radboudumc Center for Mindfulness, Radboud University Medical Center, Nijmegen, Netherlands; ^2^Kalapa Leadership Academy, Cologne, Germany; ^3^Division of Integrative Health Promotion, Coburg University of Applied Sciences and Arts, Coburg, Germany; ^4^Institute for Frontier Areas of Psychology and Mental Health, Freiburg im Breisgau, Germany; ^5^Institute of Medical Psychology, Ludwig Maximilian University of Munich, Munich, Germany

**Keywords:** meditation, self-regulation, adaptability, self-care, communication, leader self-awareness, conflict management

## Abstract

Mindfulness training is a novel method of leader development but contrary to its rising popularity, there is a scarcity of research investigating how mindfulness training may affect leader capabilities. To gain a better understanding of the potential of a new research field, qualitative research is advantageous. We sought to understand how senior leaders experience the impact of mindfulness training in their work lives and leadership ability. The sample comprised 13 leaders (*n* = 11 male) working in six organizations that completed a 10-week workplace mindfulness training (WMT). We conducted semi-structured interviews 6 to 12 months following course completion. We analyzed the data following thematic analysis steps and based on these findings, we devised a framework of the perceived impact of mindfulness training on self-leadership and leadership capabilities. We show that WMT exhibited impact on three self-leadership capacities: mindful task management, self-care and self-reflection and two leadership capacities: relating to others and adapting to change. Participants’ recounts additionally suggested effects may expand to the level of the team and the organization. We show that WMT may be a promising tool for self-directed leadership development and outline avenues for future research.

## Introduction

Mindfulness is promoted as an effective leader development tool in popular literature (Bunting, 2016; Reitz and Chaskalson, 2016; Hougaard and Carter, 2018) and increasingly offered in leader development trainings. Mindfulness has been defined as the state of “paying attention in a particular way: on purpose, in the present moment, and non-judgmentally” (Kabat-Zinn, 2011, p. 291). This state may seem at odds with the main objective of leaders, which is to get results and be future-oriented (Goleman, 2004). Lyddy and Good (2017, p. 1) showed that mindfulness may be applied to goal-directed behavior at work as a way of “being while doing.”

The process of how to develop a leader’s capacity to be effective remains a constant debate amongst scholars. Research on leader development, defined as “the expansion of the capacity of individuals to be effective in leadership roles and processes” (Day and Dragoni, 2015, p. 134) indicated that there are individual capabilities that enable leader to continuously enhance their skills in increasingly dynamic, complex and demanding environments (Uhl-Bien and Arena, 2017). Leaders who are highly self-aware, identify strongly with being a leader and are highly self-efficacious are more likely to be effective leaders (Day and Dragoni, 2015). Furthermore, researchers highlight the importance of leaders’ ability to regulate (negative) emotions. Emotional intelligence for instance, the ability to recognize and manage emotions in self and others, is related to team and leader performance (Ashkanasy and Dasborough, 2003; Ashkanasy and Dorris, 2017). There is also an ethical and virtuous aspect to managing emotions for leaders (Brown and Treviño, 2006; Jackson, 2018), a responsibility for acting in a way that causes the least amount of harm for others, grounded in an awareness that leader behaviors are strongly related to followers’ wellbeing, job and life satisfaction (Kelloway et al., 2005) and that both positive and negative emotions of leaders may be contagious. For example, increased levels of positive affect of leaders are associated with greater goal attainment and follower performance (Joseph et al., 2015). Ashkanasy and Dorris (2017, p. 78) thus argued that leaders should practice “leading with emotional labor” and learn how regulate their own feelings effectively. Finally, leaders need capabilities to effectively lead themselves. Self-leadership is defined as a set of behavioral and cognitive strategies that are related to enhanced (individual) performance (Furtner et al., 2013). This includes behavior-focused strategies such as self-goal setting and self-observation and constructive thought patterns such as evaluating beliefs and assumptions. Sampl et al. (2017) showed that a mindfulness-based self-leadership training was effective in improving self-leadership capabilities. Self-leadership is distinct but associated with active leadership styles (Furtner et al., 2013) and improvements in self-leadership aspects are thus likely to be associated to improvements in leadership style and vice versa.

There is a lack of research showing *how* leader capabilities like self-awareness and emotion regulation and self-leadership may be improved (Day and Dragoni, 2015). For example, leaders may gain deeper insight into their weaknesses and strengths by receiving 360° feedback and identifying gaps in their self-perception. Yet, the successful management of negative emotions while receiving negative feedback is a prerequisite for it to become a learning opportunity (Nesbit, 2012). Given the fast-changing nature of today’s dynamic environment, Nesbit (2012) emphasized that teaching leaders meta-skills that are needed to continuously develop themselves in a self-guided manner is a critical leadership competency. It remains unclear, however, how leaders can be trained in self-directed leadership development and thus adapt to increasingly challenging working environments *themselves*.

Mindfulness training may be uniquely positioned to provide leaders with a method useful to engage in continuous self-development by providing them with a practical tool that aids them in gaining awareness and manage their own and others’ emotions more effectively. While this is a developing research field, a variety of scholars have already laid out the potential of mindfulness training for leader development, suggesting it may impact leaders’ information processing and decision-making (Sauer and Kohls, 2011), relationship quality and communication (Good et al., 2016), ability to adapt to organizational change (Hyland et al., 2015), and even change organizational culture (Kohls et al., 2013; King and Badham, 2018). Mindfulness has been understood as a personal resource (Grover et al., 2017) that might affect leader capabilities through improvements in attention regulation, emotion regulation and self-regulation (Good et al., 2016). For example, trait mindfulness of leaders, a disposition to be mindful, was positively associated with leader psychological capital (Brendel et al., 2016) which in turn mediated the negative relation of mindfulness with negative affect and burnout (Roche et al., 2014). Therefore, mindfulness training may potentially increase the resilience of leaders. In fact, there is strong evidence that mindfulness interventions are effective in improving a number of health-related conditions including depression, pain and addiction in a clinical setting (Goldberg et al., 2018) and stress and burnout in workplace settings (Hülsheger et al., 2013; Lomas et al., 2017).

With 80% of studies set in working environments investigating the impact of mindfulness training using stress or strain as their primary outcome measure (Eby et al., 2017), the evidence-base to date is mostly limited to health-related outcomes of employees. However, scholars believe that individual mindfulness in workplace settings may be beneficial beyond stress reduction, for example when interacting with others (Glomb et al., 2011; Good et al., 2016). A recent systematic review showed that mindfulness was positively associated with pro-sociality (Donald et al., 2018). Furthermore, leaders’ trait mindfulness was positively related to external ratings of leader-member exchange quality (Reb et al., 2018), aspects of a servant leadership style (Pircher Verdorfer, 2016), and follower wellbeing (Reb et al., 2014). Finally, a workplace mindfulness training (WMT) had a moderate effect on team climate and performance in a mixed sample of employees and leaders (Kersemaekers et al., 2018). These predominantly correlational studies suggest that mindfulness training may positively affect leaders’ interaction style and their followers’ wellbeing.

Some scholars, however, have hypothesized and shown that mindfulness may have negative or unintended effects in a work context. Similar to employees, leaders could become more aware of toxic work environments due to mindfulness training which might in turn impact their motivation, performance and/or intention to leave (Walsh et al., 2017). There is experimental evidence that a brief mindfulness exercise temporally demotivated participants to perform a cognitive task, even if it was framed as pleasant. Interestingly, only task motivation but not task performance were affected by mindfulness induction (Hafenbrack and Vohs, 2018). Because participants of mindfulness interventions are becoming more self-aware, they may feel more inclined to act in accordance with their values although these might not be aligned with the best interest of an organization (Ericson et al., 2014). In two pilot studies, following MBSR training, teachers’ engagement decreased significantly compared to a control condition (Rupprecht et al., 2017) and call center employees considered leaving their job after becoming more aware of the impact of bad working conditions on their wellbeing (Walach et al., 2007).

To summarize, because mindful leadership trainings and coachings are already being offered, we need to better understand how leaders experience mindfulness training and what are the positive and negative or unintended effects. While preliminary studies suggest there is a potential for mindfulness training in leadership, research on mindfulness training for leaders is scarce and a comprehensive theoretical framework based on leaders’ experiences is lacking because “management scholars have not yet seriously undertaken that challenge” (Good et al., 2016, p. 15). Intervention studies of mindfulness in workplace context have not yet investigated the subjective perspective of leaders on how they describe self-attributed changes. They also have largely not considered workplace outcomes but rather focussed on individual and health-related variables (Eby et al., 2017). Thus, a more comprehensive understanding of the potential of mindfulness training in a leadership context and beyond stress is missing.

Our qualitative study responds to the call for a more in-depth investigation of mindfulness and leader development (Good et al., 2016). Our main objective is to better understand the impact of a second-generation WMT on leader capabilities, therefore harnessing the advantages of a qualitative research approach as a hypotheses-generating design. Providing a comprehensive overview of the effects of mindfulness training experienced by leaders may add knowledge necessary for the further development of this young research field.

### Mindfulness Training in Workplace Contexts

How may leaders increase their ability to be mindful? Both state and trait mindfulness can be developed and refined through the practice of mindfulness (Baer et al., 2006, 2012). Mindfulness interventions are usually offered in a multi-week format and require the practice of formal meditative practices and informal mindful activities, such as mindful walking, as a means to develop mindfulness. King and Badham (2018) suggested differing between first-generation and second-generation mindfulness programs. They described first-generation programs as “individualistic, therapeutic, and primarily instrumental” (King and Badham, 2018, p. 1). These interventions such as mindfulness-based stress reduction (MBSR; Kabat-Zinn, 1991) and mindfulness-based cognitive therapy (MBCT; Segal et al., 2002, 2013) were designed to alleviate stress or symptoms of depression and have been offered in both clinical and non-clinical context. Consequently, the evidence-base around mindfulness interventions is largely inferred from the efficacy and effectiveness of MBSR and MBCT on reducing psychiatric disorders or stress in healthy adults (Fjorback et al., 2011; Eberth and Sedlmeier, 2012; Kuyken et al., 2016; Goldberg et al., 2018).

However, in workplace and leadership contexts, second-generation mindfulness trainings are gaining popularity (Shonin et al., 2014; Kersemaekers et al., 2018; King and Badham, 2018). Responding to calls to re-integrate an ethical dimension central to the Buddhist understanding of mindfulness, second-wave mindfulness trainings are secular in nature but they reference Buddhist philosophy more explicitly with the objective “to produce transformational change in practitioners” (King and Badham, 2018, p. 1). The evidence-base for these trainings is to date very limited. Furthermore, the problem remains that leader development trainings in general, and mindfulness-based leader development make no exception here, are often not evaluated (Goleman, 2004).

## Materials and Methods

### Study Design and Paradigm

We conducted in-depth one-to-one interviews with participants of a WMT. We chose a qualitative design in order to gain a deeper understanding of participants’ phenomenological experience of this mindfulness training in their workplace contexts and to derive models and hypotheses from the interviews that may be tested in future studies. Our analysis was rooted in a realist paradigm assuming that participants articulated experiences and insights in an honest recount of their experienced reality.

### Researcher Statement

Following recommendations by Braun and Clarke (2006) to make the assumptions and background of researchers explicit, we present the experience and background of the authors of this study. The two lead researchers (SR and WK) have postgraduate degrees, experiential knowledge of mindfulness training and have previously engaged in both qualitative and quantitative mindfulness research. CT and PF belong to a commercial mindfulness training company that externally funded this research. CT is the co-founder of this company with a background in physics and Buddhism. He created the WorkingMind training and collaborated with a university to independently investigate the training effects both quantitatively and qualitatively. He provided the training to the leaders but had no role in the study design, data collection, decision to publish or preparation of the manuscript. PF is the data analyst at Kalapa. She holds a degree in applied health sciences and was invited to support this research. Specifically, she reached out to companies to enquire if they would be willing to participate in this research and conducted four of the interviews. Moreover, we invited a senior scientist (MW) and a professor (NK) with a background in psychology and mindfulness research to support the coding process and the completion of the manuscript. There were regular meetings between SR and WK at all stages of the coding process and manuscript completion.

### Participants

Our sample consisted of 13 leaders (11 males, 2 females) who had taken part in a 10-week WMT. Participants had a median of 5 years of leadership experience (*SD* = 4.22) and a mean age of 48 years (*SD* = 10.5). They were employed in six different organizations and had diverse professional backgrounds (six worked in technology, two in the chemical industry and five in higher or postgraduate education) (Table 1). We invited a total of 143 employees and leaders to a larger qualitative study that included employees and leaders. Out of this larger sample, we invited a sub-sample to participate in the interviews who met our inclusion criteria: (1) they completed a WMT at their organization 6 to 12 months ago and (2) they currently held a position with leadership responsibilities. Being a leader was defined as holding a management or supervisory position. Participants were invited to participate on a voluntary basis in this study via an email sent from the trainer of the course; emails were followed up by one of the researchers. We excluded interested participants if they (1) had participated in an abbreviated version of the training or (2) were not currently holding a leadership position. Because leaders and employees were recruited via an email sent to the course organizer, measures of the percentage of non-responses are not available. Participants received no compensation for their participation in the interview.

**Table 1:** Participant characteristics at time of interviews.

Interview number	Branch of industry	Interview language	Age	Gender	Years in leadership position
P1	Technology	English	56	male	10
P2	Technology	English	59	male	5
P3	Technology	German	55	male	5
P4	Technology	German	35	male	3
P5	Technology	English	44	male	6
P6	Technology	German	35	male	2
P7	Chemistry	German	54	male	4
P8	Chemistry	German	60	female	17
P9	Education	German	37	male	4
P10	Education	German	38	female	5
P11	Education	German	38	male	3
P12	Education	German	51	male	2
P13	Education	German	63	male	10

### Intervention

This WMT program named “WorkingMind” is a second-generation intervention and as such secular in nature but closely modeled after Buddhist philosophy. The training objective is to transform leader capabilities by providing insights and practical tools to increase self-awareness (view), understand and work with the contents of mind (practice) and engage with followers and teams (action). Its formal structure resembles traditional mindfulness-based interventions, but it has been tailored to meet the needs and demands of employees and leaders in a workplace environment. Compared to traditional and more clinically oriented mindfulness based interventions (MBIs) like MBSR and MBCT, the WMT differs in a number of ways (Table 2). For example, the WMT has a longer duration requiring participation in two day-long training days in addition to eight 2.5-hour-long sessions and is comprised of content and exercises relevant to leaders and workplace (Supplementary Table 1).

**Table 2:** Distinguishing features of WorkingMind training compared to typical MBIs.

Key features	WorkingMind	Typical MBIs
Duration	10 weeks	8 weeks
Group based	Yes	Yes
Average total intervention hours	Appr. 32 h (2 × 6 h day-long retreat at the beginning and end of the course, 8 × 2.5 h weekly session)	Appr. 31 (8 × 3 h sessions plus 1 × 7 h retreat day)
Number of trainers	2	1
Daily required formal home practice	10 min or more	40 min or more
Daily required informal home practice	1–2 practices per week, e.g., mindfully starting a meeting, eating lunch mindfully	1–2 practices per week, e.g., eating lunch mindfully, doing an everyday activity mindfully
Yoga exercises	No	Yes
Body scan and breath awareness exercises	Yes	Yes
Meditation on compassion and loving kindness	Yes	Optional
Guided meditation recordings and other supporting materials	Yes, app-based	Yes, recordings on CD or mp3
Psychoeducational content	Focus and attention at work, mindfully working with time, happiness, communication, collaboration and trust, self-management and leadership	Automatic pilot, awareness of body and emotions, understanding and managing stress, mindful communication
Practical exercises for mindfulness at work	Yes	No, but informal practices for integrating mindfulness into daily life
Support in integrating mindfulness sustainably in an organization	Yes	No

The intervention took place in a group setting with 12–25 participants per group and consisted of a mixed convenience sample of leaders and employees interested in the WMT with the exception of one organization where the group consisted solely of leaders. Participants learned a variety of formal and informal meditation practices including mindfulness meditation, walking meditation, pausing meditation, body scan, and compassion meditation and were asked to practice for at least 10 min daily. Furthermore, participants were encouraged to practice mindfulness in everyday life (informal practices) which included applying mindfulness in conversations at work (listening, dialogue) and in team meetings (a minute of silence before a group meeting), noticing positive experiences, using email in a mindful way and daily journaling. Participants received app-based audio recordings of formal and informal mindfulness practices. Moreover, the WMT’s comprehensive psychoeducational component included information and discussions about the neurobiology of stress and emotions, the functioning of attentional networks, mindful task management and mindful team collaboration (Supplementary Table 1). The intervention was delivered by two experienced trainers with 10 or more years of personal practice and prior experience in leading group processes in a company setting and a good understanding of the relevant neuroscientific background.

### Data Collection

Two researchers fluent in both German and English conducted the interviews either face-to-face or over the phone which lasted between 25 and 65 min. Three interviews were conducted in English and the remaining 10 interviews were conducted in German. At the start of the interview, interviewers collected some demographic data and their average weekly mindfulness practice during the course and in the last 4 weeks. Participants were informed that the interviews’ objective was to learn more about their “positive as well as negative experiences of the training and of bringing mindfulness to your professional life.” Using a semi-structured topic list, the interviewers started with open-ended questions and, based on responses, prompted for additional details and examples (Table 3). The interviewer had the freedom to pursue unexpected areas of interest and ask for more detail. All interviews were transcribed in the language they were conducted. We conducted interviews until we couldn’t identify new themes and agreed that data saturation was reached. This study was part of a larger quantitative study for which IRB approval was granted and written informed consent was obtained from all participants (Kersemaekers et al., 2018). Participation in this study was voluntary and all participants gave informed consent prior to the start of the interview.

**Table 3:** Topic list of semi-structured interviews.

• Overall, what was your experience with the mindfulness training?
• Did the training affect your leadership? If yes: How?
• Other aspects (If not already mentioned):
Did you experience any change in …
… the way you manage your work load?
… the way you organize work within your team?
… the way you collaborate with others?
… in your longer-term vision?
Did you experience any negative effects of the training?
• How did the training contribute to these changes?
• How feasible was it for you to take part in the training?

### Data Analysis

We followed the thematic analysis steps as outlined by Braun and Clarke (2006). We analyzed the data using the qualitative research software Atlas.ti. We approached the interviews and analysis with a basic understanding of the potential impact of mindfulness training in mind that was refined in the process of leading the interviews in an ongoing reflexive dialogue about the material.

#### First Stage – Familiarization With Data

Two researchers transcribed the digitally recorded interviews and reread the transcripts to familiarize themselves with the data.

#### Second Stage – Initial Coding of Data

Based on the interview transcripts, interviews were first coded in English by two researchers independently to minimize subjectivity. Following that, the researchers compared and discussed the codes until they reached a consensus.

#### Third Stage – Finding Themes

In this stage, the codes were reviewed and discussed in order to define an initial coding taxonomy. All coders and two additional independent researchers not prior involved in this study participated in the process of finding themes. By reviewing the codes organized in groups, the external researchers, assisted by the researchers familiar with the data, created preliminary themes and subthemes out of the codes and groups of codes presented to them.

#### Fourth Stage – Reviewing and Naming Themes

In a further refining process, we compared the themes and subthemes with the original quotes. Quotes which received multiple codes were carefully reviewed and attributed to the theme or subtheme that appeared most relevant. We also looked for originality and depth of a leader’s account; thus, we included quotes if they provided a rich description of the perceived impact (Braun and Clarke, 2006). During this procedure, links between themes became eminent and subthemes were defined until agreement on a final framework was reached. To further reduce the amount of themes, we focused on responses that were related to perceived effects of the WMT on shifts in the leaders’ mental responses and behavior at work.

#### Fifth Stage – Completing and Revising Manuscript

Themes and subthemes were named based on the content of the quotes and our understanding of their relation to operationalizations in the field of leadership as well as mindfulness research. These names were further refined in the review process to minimize overlaps with other constructs.

## Results

### Framework of the Perceived Impact of WMT

**FIGURE 1:**
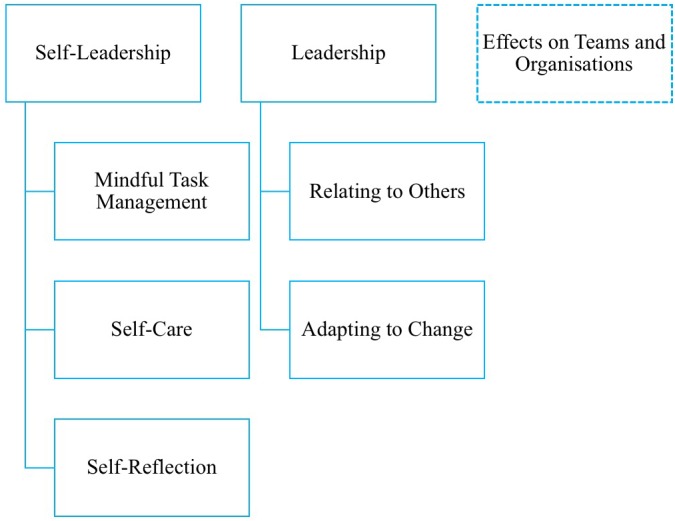
Framework of the effects of WMT for leaders.

Figure 1 displays a framework of the outcomes of the analysis. Leaders indicated that the training impacted the development of self-leadership and leadership capacities. Additionally, leaders reported occasional spill-over effects to their teams or to the organizational level. This was an interesting emerging topic which we will briefly summarize at the end of the result section. It is important to note that none of the interviewees mentioned experiencing any adverse effects to WMT – even after additional prompts from interviewers.

### Effects of WMT on Self-Leadership

Leaders’ improvements in self-leadership were evident in new skills concerning effective task management, self-care and self-reflection. Our data suggests that the leaders used mindfulness to enhance their effectiveness and performance including becoming more aware of their personal limits of performance. Specifically, enhanced levels of self-awareness, attention and emotion regulation were applied as mechanisms of change to improve their effectiveness.

#### Mindful Task Management

##### More single-tasking

Leaders acknowledged that dynamic work environments often challenge their ability to perform focused work and some recalled a limited ability to sustain concentration prior to the start of the training. Rooted in a greater ability to regulate attention, leaders indicated setting the goal to “focus more on individual tasks rather than multitasking” and having a greater ability to observe themselves in order to complete a task first before starting with the next.

“I try to work through mails in a concentrated way and really finish writing them without doing anything else. This really created added value for managing my daily work load.”

##### Managing distractions

Leaders introduced new measures to actively cope with a multitude of distractors in their work life. Many leaders reported reducing the amount of potential distractions, e.g., in the service of being better able to single-task. A major source of distraction was attributed to automatic notifications from emails and phones and some leaders indicated limiting and controlling access to the respective devices:

“I turn off all automatic messaging, I even turn off my phone or office chat so that I can’t be disturbed during a challenging work task (...) I used to receive emails day and night and even check them in the evening on my couch. (…) This is a small change that gave me an enormous life quality back.”

Because participants were better able to observe where their attentional focus wandered, some changed the conditioned automaticity with which they interacted with their devices and information channels and “deliberately chose that no, I don’t want [to look at my phone] anymore, I put it away now.” While adopting new strategies to handle constant access to information are not specific to leaders, leaders also indicated that their behavioral pattern in response to task interruptions by followers had changed. Instead of automatically reacting to a followers wish to speak to them, they used mindfulness skills to pause and discern whether they needed to immediately shift their attention or whether it would be favorable to schedule a formal meeting, taking into account both their own emotional state and that of the follower (Table 4).

**Table 4:** Themes, subthemes and illustrative quotes of the effects of WMT on self-leadership.

Theme	Subtheme	Illustrative quote
**Mindful task management**	More single-tasking	It made me focus more on individual tasks rather than doing multitasking. I still tend to do multitasking, I still do have that distraction. But it’s not as strong as it was before I started with meditation. (...) I mean in a sense meditation has focused me a little bit more on spending more time on individual tasks and making sure they are complete.
	Managing distractions	I take greater care when a colleague comes and asks if I have time for a conversation to assess if that’s actually a good time for a conversation and if I am capable of concentrating fully on it. It happened more often that I responded: Maybe in half an hour and that we agree on an appointment. Before, when I had time in principle, I would lead the conversation right away which of course interrupted my own work and I was often not fully present in the conversation.
	Conscious transitions	When I am going from one appointment to the next, I am taking a bit more time – which I have done rarely before – to arrive 5 min earlier. Simply to take in the atmosphere of the new place (...) When I have to switch in my head between one topic and the next, I am taking more time to transition to the next one.
**Self-care**		I now realize more that I am exhausted – that is, a mental and physical exhaustion – and then I enquire whether we can resume the next day, if it is not absolutely urgent.
**Self-reflection**		Mindfulness brought along with it a better common understanding about (...) how one reacts, but more importantly for me, how one was perceived. (…) [For example, I realized that] I was always too forceful. I was not being able to see the indications that I was being too (...) offensive.

##### Conscious transitions

Respondents gave examples of using mindfulness as an anchor in transitions, e.g., between work and home or between two work tasks, meetings etc., These spaces between two tasks were used to become aware of the current state of body and mind or to pay attention to the outside world. One respondent installed a regular mindfulness break in order to cultivate the transition from home to the start of the work day:

“I meditate right when I arrive at the office (..) to clear my head (...) and to be able to concentrate on the first work task, to familiarize myself with my work day and to organize myself.”

Thus, the meditative practice was employed in order to be more effective at work. Another leader often had to walk to different locations at his workplace to attend and lead meetings and used the transitional period to practice mindful walking awareness, again in order to be better prepared for the next meeting:

“When I have to go from one location to another, I now pay attention to the way I am walking and to my inner state. So it’s not only about where I need to go but also the way. (...) It clears my head for what I do next or to let go of what happened before.”

#### Self-Care

An element of greater self-care emerged in the leaders’ accounts as they became more aware of their personal limits and simultaneously more allowing to respond to limits by choosing to postpone a work task and take rest when they assessed that their ability to perform was low.

“When I had a strenuous long appointment, and then a colleague comes in and says, ‘I would like to discuss something important again,’ I now realize more that I am exhausted – that is, a mental and physical exhaustion – and then I enquire whether we can resume the next day, if it is not absolutely urgent.”

It may be argued that self-care is an effective self-leadership tool to maintain high effectiveness. This includes acknowledging that a person needs to take a break:

“So it was probably the first time in many, many years that I decided I needed a 4-week break this year and during these 4 weeks I didn’t do any meditation, I just took a break of everything. And that was very useful in the sense that I had a sort of reset (...) coming back to work I felt much more motivated, a lot more energized to carry on the tasks.”

Leaders also gave examples of sharing their emotional state more readily with followers, which may be regarded as a way to model self-care to others. Interestingly, one participant acknowledged that becoming more aware of his limits contributed to working less:

“I am more aware of myself and my limits. So I noticed, for example, that I work less. (...) I can get out of here better, although things aren’t finished yet, because I just say to myself, ‘Ok, it’s enough for me today and it’s not good for me to be here any longer and it wouldn’t be as effective anymore,’ to be honest.”

#### Self-Reflection

Through greater self-observation and detachment from negative emotions, leaders indicated gaining insights into assumptions and beliefs and reflecting on how their emotional state and behavior affected the reactions of their colleagues. This subtheme is an intraindividual mental process with great relevance to leadership behavior which overlaps with the leadership theme “relating to others.” Its distinguishing feature is that the leaders *internally* reflect on previous behavior, set a new goal and reappraise situations that they formerly perceived as stuck. One leader reflected on expressing his anger to followers.

“Basically, if you sit and scream and shout all day long because you are not getting any feedback, you are not encouraging team work in a sense and then you know at a certain point that something is wrong.”

He further described testing different approaches to getting the feedback he needed in a more effective way. Another leader took a feedback from a colleague to heart:

“Recently, a colleague presented something and asked me for feedback. I reviewed it and found it quite good (...). She responded that she’d never expected [a positive feedback] from me and that she was really happy about that. I must have left quite a negative impact on others if they are so surprised that I give them positive feedback.”

At the time of the interview, he was actively seeking out opportunities to acknowledge positive performance of his team more readily. Finally, one leader described seeking feedback more readily and using it more consciously to reflect on leadership behavior:

“I can cope with feedback in a much more calm or composed way and that maybe why I am also asking for feedback more often and more consciously.”

### Impact of WMT on Leadership

Leaders were using mindfulness skills, specifically self-regulation and emotion regulation, to continuously develop and evolve both as leaders and actively shape and form their formal and informal role. The leaders described both mental and behavioral shifts, often but not always with the former paving the way for the latter. For example, leaders gave comprehensive examples of how they changed their perception of others, and in turn related differently to them. We grouped examples of mental shifts under leadership if they related to leadership situations. Following WMT, leaders recounted many instances where they perceived themselves as engaging in a more mindful way with followers. For example, they indicated that they listened in a more mindful way and that they experienced a greater ability to regulate their emotions and guide followers through emotional difficulties. Leaders also recounted a greater acceptance of change and ability to focus on solutions (Table 5).

**Table 5:** Themes and illustrative quotes of effects WMT on leadership.

	Themes	Illustrative quote
**Relating to others**	Listening mindfully	Rather than trying to impose my opinion or approach, I started listening to what people said and how people actually reacted.
	Buffered emotional reactivity	And I’ve noticed that one lets himself emotionally be triggered [in tense meetings]. I was able to become more aware of these emotions and to say [to myself] “Okay, I am not letting myself get involved in this emotional game (...)” or to say “This emotion is partly justified and should be displayed (laughs).” That’s a much more conscious decision now.
	Being less judgmental of others	I am better able to let others have their own opinion. There is not only one truth, not only one way of looking at problems. And that also has something to do with tolerance. (...) I have become a little more sensitive for this.
	Lower degree of self-involvement	I take work seriously but I don’t take myself so seriously anymore.
	Heightened awareness of followers’ needs	I noticed that I went from “I could get upset, get angry about it now” to a mode that I was actually trying to help him get out of the situation as quickly as possible. That was a new experience.
**Adapting to change**	Accepting change	I am more able to accept situations [at work] that I can’t change anyways. So I do not get so worked up about circumstances. And my emotional state is not so dependent on others’ actions or the environment. There is a certain equanimity.
	Focus on solutions	I feel more able to see (...) possible solutions. I think I have gotten a wider perspective and see more opportunities or leverage points where we can assert our interests.

#### Relating to Others

##### Mindful listening

Practicing mindful listening was encouraged during the WMT and most leaders commented on the lasting effects of this practice. This leader stated that mindful listening has become her new default mode of listening:

“[Learning about] mindful listening was very important. Not only practicing this during the training but also applying it afterward. I am enthusiastic to see the results I get when I focus on practicing that [mindful listening]. I think [I] even developed a kind of routine, thus every conversation feels more intense, more mindful or more attentive.”

Another aspect of mindful listening was a greater ability to sustain attention in meetings and conversation highlighted by an element of choice. This ability was irrespective of the personal interest in a topic, e.g., one leader gave an example of a presentation he regarded as boring but had to attend. Instead of thinking about other tasks, he chose to stay present and experienced the quality of the presentation as more nuanced. Leaders observed that they were taking greater care to listen more attentively to others as well as becoming more aware of their own and others emotional states so that “when speaking with your team members, [I] really speak with them.” Some also recounted giving others more space in theirs discussions and noticed a reduced need to comment on everything:

“Rather than trying to impose my opinion or approach, I started listening to what people said and how people actually reacted.”

Finally, the ability to listen mindfully was perceived to be related to a heightened ability to see things as they truly are:

“I try to (be mindful) when discussing with somebody, especially when there is a tension with the subject, the situation or the person. I try to listen mindfully, to look at the face, to see what is actually happening, to try not to build up with my own imagination and assumptions, extrapolations of things, just to stay on the facts.”

##### Buffered emotional reactivity

Leaders described applying a new set of emotion regulation strategies to leadership situations. By gaining both greater awareness and greater detachment of personal and others’ emotions, they claimed to have learnt to be less reactive to emotions in interaction with others. Leaders gave examples of being less easily triggered by emotions of others and experiencing more equanimity in encounters with followers in general. Some leaders described that they had perceived an enhanced ability to regulate their emotions even in tense situations and choose their emotional response:

“Meetings can become quite tense when interests are explicitly expressed. And I’ve noticed that I let myself emotionally be triggered by that. I was able to become more aware of these emotions and to say [to myself] ‘Okay, I am not letting myself get involved in this emotional game (...)’ or to say ‘This emotion is partly justified and should be displayed (laughs).’ That’s a much more conscious decision now.”

In general, leaders felt better equipped to respond to emotionally difficult situations, be it with regard to their own or their followers’ emotions. One leaders concluded that she better understands “how people react and why they react that way.” Leaders felt less “afraid” or defensive about these experiences of shared emotions and responded with greater openness, drawing from other skills such as mindful listening and staying present. For example, one leader noticed that he was better able to handle negative emotions of followers:

“I realized when you see [an emotion] with a certain detachment, you see that maybe it’s a small thing and also that the emotional reaction of the person is not such a big disaster. We overreacted, we are human, what this person is feeling is a small thing, we can live with it. It’s about re-dimensioning it [the negative emotion] so that it is less scary and less troubling.”

In sum, leaders that noticed being less emotionally reactive following WMT saw a great benefit for their leadership practice, not only for themselves but also for guiding followers through emotionally charged situations. This leader described the summarized the effects of being less emotionally reactive in leadership situations:

“[Leadership] changes because you don’t escalate the situation. Somebody comes in that is very angry about something and then you react because his anger makes me angry and then it becomes bigger than it needs to be. But instead if I don’t escalate it and I am more attentive to what this person is communicating you start to (...) listen and little by little, it doesn’t always work out, but maybe the person starts to listen to you as well and abandon a bit the emotional state and you come a bit more to the factual things.”

##### Being less judgemental

The leaders suggested that they became more aware of the prejudgements they ascribed to followers and how that impacted their behavior toward them. This leader describes how he became aware of prejudging team mates:

“[My team and I] spend a lot of time together in the same space and after a while you start to build a sort of prejudgment about people about how they would do something or how would regard to something and issues would develop. That is something at least on my level, I try to do less. I do not manage it completely not to do it at all, (...), but I try to change it.”

Another leader noticed that his tendency to “judge others and putting people in [categorical] boxes decreased. I am more attentive and open now.” And another one noticed that by assuming that a female colleague had a hidden agenda he was unable to listen to her but became more aware that his judgment of this person may be “not true.”

##### Lower degree of self-involvement

This subtheme was created to describe a sometimes subtle shift of the perception of a self following WMT, characterized for example in meetings as a shift from feeling the urge to contribute to granting others more space. Leaders gave examples of taking others’ behavior less personal, which resulted in a lower inclination to perceive issues as related to themselves.

“I take work seriously but I don’t take myself so seriously anymore and I don’t take what happens so seriously. So it’s clear that my work is still fundamental and I approach it with seriousness but I don’t make a big deal out of things.”

Being less self-involved allowed leaders to empathize with others more (Table 5). Taking oneself less seriously lead for this leader to the realization that most incidences have “(…) nothing to do with me. Rather than always feeling self-pity or having the impression that the whole world was against me, I turn that around and start understanding, actually, it’s not [about me] at all.” By becoming more aware of the tendency to take things personally this leaders went on to mentally challenge his assumption, to check whether it is actually to true or not. Another leader shared that by taking himself less seriously, he felt more content and open toward others. This opened up mental space for a heightened awareness of the needs of followers.

##### Heightened awareness of followers’ needs

Based on a reduced involvement with themselves and an increased awareness of others’ emotions, some leaders described a greater ability to identify and serve the needs of their followers. This leader described becoming more interested in a follower’s emotional state:

“I try to be more aware of my followers’ emotional or physical state and to consider that in my respective actions.”

Following that, this awareness enabled leaders to shift the focus on identifying helpful ways to support a follower in a specific work situation. This included becoming aware of incentives of followers and adjusting actions accordingly to better meet those needs. In one example, a leader was confronted with an intern who had just experienced a family tragedy. He concluded that it was in the intern’s best interest to take some time off even though this would cause significant delays in his project. Leaders also indicated becoming more aware of the appropriateness of a formal environmental setting in a discussion. For example, in a conversation in passing that turned into an emotional discussion, a leader suggested to move the conversation to a different setting to safeguard the person.

#### Adapting to Change

Some of the interview examples suggested a greater agility in responding to change and the resulting challenges it may pose. The accounts indicated two consecutive subthemes: accepting change and focus on solutions.

##### Accepting change

Leaders indicated that they were more embracing and accepting of changing situations, caused by a deeper understanding that change happens and that it’s not useful getting worked up about it. They were more prone to accept things that can’t be changed in the first place, such as an important delivery that has been postponed or structural changes in the company.

“This new information meant that a project that I wanted to move forward would come to a standstill. And I think I would have taken that much more to heart in the past. It would have annoyed me and that wasn’t the case at all in this case. I accepted it relatively quickly that we will have to interrupt the project.”

There was a greater tendency to embrace changes that were necessary but may also pose additional risks. In one leader’s example, the awareness of having developed improved coping skills to handle distress innate in such an endeavor supported him in managing a challenging technical restructuring:

“If I imagine myself taking that sort of responsibility before I had done the course, it would have costed me a lot of lost nights of sleep. Now, not a single one.”

##### Focus on solutions

It’s important to note that acceptance wasn’t related to a greater passivity but made way for a greater focus on solutions, which replaced a tendency to get worked up about change or worry about the outcomes. Leaders described a better ability to cope with change by focusing on alternative and creative solutions:

“I am informed that the delivery that is supposed to come now is delayed by 2 months. Before I would have been extremely concerned, angry about it. Now, I approach it in a completely different way. I try to understand, why this has happened. And next, I try to understand what is going to happen because of the delay and what we need to do to adapt to it. Afterward I look to see if there are chances that this could happen again in the future and if so, if they can be avoided. And if not, how can we adapt to it. In a way, it’s very systematic and more detached, I don’t let it get to me like before. But in the end it’s much more satisfying and (...) so far, it’s been more effective.”

This also extended in one case to a greater commitment to the organization and its goals by looking for solutions:

“It’s become a stronger necessity for me, to say, something is not right here. It’s not [in my area of responsibility] but (...) I have an idea how it can be improved and then I take action. Before, I thought more often that I don’t care. But I do care now.”

### Spill Over Effects on Team and Organizational Processes

Leaders indicated that mindfulness training of various team members had the potential to shape team meetings and even ignite change of organizational processes. Various leaders reported that they found it important that fellow leaders or team members took part in a training. First, to share an experiential platform about mindfulness was regarded by one leader as having “a totally different basis for communicating with [other team members]” which can be applied in a conflict situation with a team member “to find another level of discussion” by reminding each other “to speak mindfully about that.” Second, leaders attempted to change team communication. Teaming up was regarded as crucial by this leader based on the fear that:

“If I am on my own in a bigger group of people trying to [apply these new practices] will people make fun of me? But when I am with a team of ten people and there are two of us who (...) lead the conversation mindfully (...) that makes it much easier.”

She teamed up with another former participant of the WMT to introduce mindfulness in team meetings with the goal to change team climate and effectiveness.

In another strong example, a whole mindfulness group became more curious about automatic processes that they felt had negatively impacted their ability to focus on their work: The need to attend many long meetings. They decided to attempt to change the system.

“We started wondering why our meetings had lasted as long as they lasted and developed the theory that this is due to the meeting software suggesting a meeting lasts 1 h. Everyone accepts that without giving it too much thought. Our idea was to change the system so that it will now suggest 30 min [meetings]. If you need more time, you will have to actively think about it. But that takes effort (...) so in effect the conscious thinking will be turned on. (...) It took a while to get this through but now we’ve launched it [on a company-wide level].”

## Discussion

Our results shed light on the multiple ways in which WMT developed leader capabilities and, crucially, that leaders continued to apply mindfulness skills 6 to 12 months after completing WMT. Although research has consistently pointed to the importance to develop capacities like self-observation, self-regulation and emotion-regulation for both self-leadership and leadership development (Nesbit, 2012; Day and Dragoni, 2015; Ashkanasy and Dorris, 2017), there is a scarcity of research investigating how leaders can develop these skills. Mindfulness research on the other hand has been able to demonstrate the impact of mindfulness training for attention and emotion regulation but there have been mixed predictions about its potential in work environments and for leaders (Reb et al., 2015; Good et al., 2016; Walsh et al., 2017).

This is to our knowledge the first study showing that self-assessed self-leadership and leadership capacities can be improved with WMT. The story participants tell is that mindfulness impacted the way they behave and think at work in terms of how they lead themselves and others. We found improvements in three self-leadership capacities, i.e., cognitive and behavioral strategies to increase performance (Furtner et al., 2013): mindful task management, self-care and self-reflection and two leadership capacities: relating to others, and adapting to change.

*Mindful task management*: Previous research has demonstrated that mindfulness training affected attention regulation including attentional efficiency and attentional control (Good et al., 2016) but research has not investigated if and how increased attention regulation benefits task management. Our data suggests the ability to manage tasks more effectively improved by becoming aware of what prevents effectiveness and consequently adopting new strategies. Our findings do not support Dane’s (2011), p. 1005) theoretical assumption that mindfulness may attribute to misallocating “attention toward potentially trivial stimuli,” instead our findings suggest that the effects seems to be related to increased self-regulation (Brown et al., 2007): Leaders set a goal, e.g., to stay on a task, reduced distractions and monitored goal attainment.

*Self-Care*: Participants became more aware of personal limits and their need for self-care. In practice, they increased their ability to discern whether prioritizing self-care over continuing to work would be most useful in situations where they noticed exhaustion. This was at times reflected in a different relationship to work. For example, one leader indicated having a greater connection to his general motivation in life buffered his willingness to overwork. This result may be viewed as a negative outcome of this training associated with a lowered motivation or engagement at work (Hafenbrack and Vohs, 2018). Greater self-care may alternatively be regarded in light of a more effective use of personal resources which may eventually prevent *presenteeism*, which is more prevalent in higher-paid staff such as leaders (Goetzel et al., 2004). We found support for both hypotheses in our data and it seems likely that additional variables, e.g., external moderators, are better suited to explain the relationship between self-care and engagement (Rupprecht et al., 2018).

*Self-reflection*: Leaders showed that the greater self-awareness contributed to self-reflection, a key outcome in leadership development which was related but distinct to other themes and subthemes. For example, being less reactive to emotions in leadership situations was a necessary prerequisite to becoming more self-reflective. Leaders described situations such as a greater readiness to seek feedback because they felt better able to handle negative emotions which Nesbit (2012) argued may aid leaders to use feedback for self-guided leader development.

*Relating to others*: A main contribution of this research is that WMT may affect the way leaders relate to others, in particular they seem to be better able to modulate intrapersonal cognitions and emotions in communication. The emerging mental and behavioral shifts the leaders described suggested an increase in relationship quality which is in line with correlational research showing that leader mindfulness was positively related to LMX quality and that mindfulness intervention for couples increased relationship quality following training (Carson et al., 2004; Reb et al., 2018). Our results indicate that mindful communication skills such as mindful listening may facilitate greater relationships which is in line with a recent study showing that employee rated mindfulness in communication mediated the effect of leader mindfulness on employee satisfaction (Arendt et al., 2019). The results further indicate a potential change to a more desirable leadership style. For example, the greater focus on building a supportive relation to followers potentially indicates a shift to a human-oriented leadership style which is characterized by great supportiveness and little aggression (de Vries et al., 2010). Interestingly, the subthemes we unearthed in this theme are all inter-related and mutually enhancing. For example, listening mindfully to followers was related to being less reactive to emotions which in turn seemed to contribute to decreased self-involvement of leaders and a less judgmental attitude. Participants reported becoming more aware of their own and others’ emotions and being better able to modulate their emotions in a more sensitive way according to the needs of a leadership situation. Ashkanasy and Dorris (2017, p. 78) argued that because of their unique position to (de-) motivate others, leaders should engage or “lead with emotional labor,” that is they should become aware of emotions that are conducive to motivating others and able to effectively regulate their own emotions in the service of this goal. Our findings are in line with studies showing that MWT enhanced positive emotion regulation strategies of employees (Hülsheger et al., 2013) and can decrease bias (Kang et al., 2013). Our results expand on this by showing that participants were better equipped to *guide followers* through emotional experience by showing empathy and deescalating a tense situation (Thiel et al., 2015). Participants appeared less self-involved, which may signify a greater ability to express humility, “a person’s tendency to approach interpersonal interactions with a strong motive for learning through others” (Owens et al., 2013) or a decreased self-concern (De Dreu and Nauta, 2009). However, the construct presented in these interviews may also go beyond these constructs and suggest that the perception of a self shifted toward a genuinely less self-involved point of view. This change of perspective of a self has been demonstrated in neuroscientific research of mindfulness training as a decrease of self-referential processing (Hölzel et al., 2011) and recently Reb et al. (2015, p. 21) suggested reduced “ego involvement” might be linked to a leader’s motivation to perceive wholesome rather than unwholesome goals. In a post-heroic era where leader humbleness appears more desirable, WMT may guide them in making that shift in an authentic and experiential way. In the accounts of the leaders, a decrease in self-involvement seemed to be related to a heightened awareness of followers needs, though research has shown that self-concern and other-orientation are two distinct constructs (De Dreu and Nauta, 2009). A heightened awareness of followers’ needs may also be related to the development of a servant leadership style (Pircher Verdorfer, 2016) or specific follower characteristics, e.g., followers may experience greater psychological need satisfaction (Reb et al., 2014).

*Adapting to change*: Finally, fostering organizational adaptability is a major goal of leader and organizational development (Uhl-Bien and Arena, 2018) and scholars are calling for the development of more agile leaders (Doz and Kosonen, 2010). Our interviews indicated an increased adaptability to change by accepting change and focusing on solutions thus leaders may be better equipped to enable others to thrive in dynamic situations. A supporting finding is that leaders reported a greater ability to manage and embrace risks. While a previous intervention study found no effects of a mindfulness training on tolerance for ambiguity of leaders (Brendel et al., 2016), the changes following WMT were characterized by less automaticity and greater agility which indicate leaders may have become more adaptive to the demands of their profession. Thus, our results suggest that this mindfulness training might contribute to the development of positive organizational behavior, such as resilience (Luthans, 2002) which is in line with correlational findings showing that trait mindfulness was positively associated with resilient behavior (Malinowski and Lim, 2015). However, our findings may suggest that rather than merely coping with unexpected changes leader were better equipped to truly accept and anticipate them and consequently look for the most useful response or solution. This may be the ground in which greater innovation and enabling leadership behavior can flourish.

It seems critical, however, for leaders to keep engaging in regular formal mindfulness practice as the accounts of these leaders were richer than those who stopped practicing meditation. Leaders who reported not practicing meditation or mindfulness anymore at the time of the interviews gave generally fewer examples of the impact of the training. Interestingly, the self-reported amount of meditation practice in the last 4 weeks was significantly positively associated to the number of codes of the perceived impact of the training per participants (*r* = 0.55, *p* = 0.03) (Table 6). This indicates that the depth of the perceived and sustained impact of the training is higher when leaders continue practicing meditation; a finding previously shown in a systematic review indicating a positive relationship between self-reported home practice and intervention outcomes (Parsons et al., 2017).

**Table 6:** One-tailed correlation between mindfulness practice and number of distinct codes.

Correlations			

		Practice minutes	Number of codes
Practice minutes^†^	Pearson correlation	1	0.559^∗^
	Sig. (1-tailed)		0.029
	*N*	12	12
Number of codes	Pearson correlation	0.559^∗^	1
	Sig. (1-tailed)	0.029	
	*N*	12	12

*^∗^Correlation is significant at the 0.05 level (1-tailed). ^†^Self-reported average weekly mindfulness practice in the last 4 weeks.*

To summarize, we contribute to the theory and research of leader development in three important ways. First, a main contribution of this research is that WMT can be an avenue to self-directed leader development (Nesbit, 2012). Nesbit postulated that rather than keeping up with new leadership trends, the focus should shift on giving leaders the skills to “take greater control of their development.” He identified three meta-skills: self-reflection, managing emotion reactions to feedback and self-regulation. Leaders recounted examples for all three of these categories and showed that they often went beyond self-understanding to test or implement self-change. WMT seems a promising intervention for leaders to increase self-awareness – be it awareness of the way they manage their tasks, lead a tense conversation or react to change (Day and Dragoni, 2015). Typically self-awareness of leaders is increased by 360° feedback tools, yet, our results show that mindfulness training like WMT might additionally prepare leaders to better manage negative emotions to feedback (Nesbit, 2012). Mindfulness training in general is conducive to becoming aware of mechanical reactions and the accounts of WMT in particular showed that leaders gain insights into their self-views and became aware of automatic reactions. Their reports further indicated that they continued to do so 6 to 12 months post-training completion, suggesting, that they developed sustainable meta-skills that they continued to use to develop self-leadership and leadership.

Second, our results give insights into the black box of how mindfulness training may shape leader behavior and even leadership style. Our data shows that there is a potential for mindfulness training of leaders that goes beyond decreasing stress and improving resilience. As highlighted above, the themes we created appear to overlap with multiple constructs and operationalizations of leadership capabilities and leadership styles such as the servant, authentic, human-oriented and agile leadership style. Thus, we contribute with this study to the development of hypotheses regarding the potential impact of mindfulness training on leadership capabilities and leadership style. Scholars may also be interested to test whether mindful leadership is conceptionally distinct style of leadership characterized by greater focus on mindfully relating to others and adaptability to change.

Third, our findings show that mindfulness training may increase self-leadership capacities which is in line with a previous study of the impact of a mindfulness-based self-leadership training (Sampl et al., 2017). Self-leadership is distinct but positively associated with active leadership styles (Furtner et al., 2013) and improvements in self-leadership aspects are thus likely to be associated to improvements in leadership style and vice versa. Similarly, we found various cross-over points between self-leadership and leadership themes which seemed to mutually enhance each other.

### Practical Implications

A primary goal of leader development research has been to improve leader effectiveness (Day and Dragoni, 2015). Unearthing the potential of this mindfulness training for leaders and examining its role in improving self-leadership and leadership has implications for practitioners. WMT may produce long-lasting mental and behavioral shifts in leaders and thus may be a promising intervention for self-directed leader development. Leaders were motivated by workplace-specific program elements suggesting that it’s useful to develop novel mindfulness-based trainings for leaders. However, more quantitative research is needed first to support the preliminary findings of this study.

### Limitations and Future Research

We carefully considered each step of this research and followed the stages of analysis for qualitative research as outlined by Braun and Clarke (2006). The leaders participating in this study completed a WMT which is similar to standard mindfulness-based interventions (MBIs) but also differs to it, e.g., in terms of content, workplace applicability (Table 2). Future studies could compare different training types and a control condition to assess the specific effects of training components on leader capabilities.

Our sample is self-selected because participation in the interviews had to be voluntary. Even though we emphasized that we are interested in all kinds of experiences, the purposive sampling strategy might have attracted a specific subsample of participants. Thus, the results presented here might be skewed toward an overly enthusiastic view of the potential of mindfulness for leadership. This hypothesis is perhaps supported by a particularly low participation rate in a team with an ongoing relationship conflict that resulted in a reportedly tense group situation. It is likely that the group climate may have influenced the experience of and involvement with the training. To better understand potential moderators, future qualitative studies could specifically investigate the impact of WMT and other MBIs in different team climates. Because 84% of our interview participants were male and men are typically underrepresented in qualitative and quantitative mindfulness research. The low number of female leaders was expected in this sample considering that (1) we recruited leaders in mostly male-dominated branches of industry and (2) women are typically underrepresented in leadership, e.g., only three in 10 leaders are female in Germany (Holst and Friedrich, 2017). This sample provides new insights into what motivated predominantly male leaders to take part in a mindfulness training and how they experienced it. While we consider this a strength of our study, the experience of mindfulness training may differ for female leaders to some extent and future research could evaluate whether there is a gender effect.

Our findings suggest that WMT can positively impact leader-follower-relations at work. Apart from quantitatively assessing these effects with leader self-reports, future research should additionally integrate leader-follower dyads and whole teams to increase the reliability of these findings. 360° feedback tools can help to assess whether the mental and behavioral shifts of leaders can be noticed by others and impact others.

Finally, we invoked recent theoretical developments that showed mindfulness can be regarded as a team-level variable (Yu and Zellmer-Bruhn, 2018). While this study focused on the impact of mindfulness on self-leadership and leadership, we encourage scholars to investigate how leaders apply mindfulness skills to affect change on a team and organizational level. Leaders reported some spill-over effects which indicate that mindfulness training may be more far-reaching than the leader-follower-dyad.

## Conclusion

We add to the increasing interest in mindfulness for leaders by showcasing the leaders’ perspective of the potential of this WMT. Our outcomes suggest that leaders improved three self-leadership capacities: mindful task management, self-care and self-reflection and two leadership capacities: relating to others and adapting to change. The effects of WMT may also expand to the level of the team and the organization. We hope our research sparks more nuanced research of the impact of mindfulness training for leader development.

## Ethics Statement

This study was part of a larger study for which IRB approval was granted by Coburg University of Applied Sciences, Coburg. Participation was voluntary and participants consented prior to the interview recordings.

## Author Contributions

WK and SR designed and planned the study, and interpreted and grouped the data. SR and PF collected the data. SR, PF, and WK coded the data. MW and NK supported the theme and subtheme finding process. SR drafted and revised the manuscript. WK, MW, and NK provided substantive suggestions for revisions and critically reviewed subsequent versions of the manuscript. CT developed and provided the WMT. All authors reviewed and approved the final manuscript.

## Conflict of Interest Statement

The research was funded by Kalapa Leadership Academy. CT is the co-founder of Kalapa Leadership Academy, provided the WMT and had no role in the study design, data collection, and decision to publish or preparation of the manuscript. PF is employed as a research consultant by Kalapa Leadership Academy, involved by the research team in conducting interviews and coding interviews. WK and SR were funded by Kalapa Leadership Academy to independently conduct this study. The remaining authors declare that the research was conducted in the absence of any commercial or financial relationships that could be construed as a potential conflict of interest.
