# Supplementary material for: Mindful Leader Development: How Leaders Experience the Effects of Mindfulness Training on Leader Capabilities
Source: Front Psychol. 2019 May 15;10:1081. doi: 10.3389/fpsyg.2019.01081 (PMC6529524; doi:10.3389/fpsyg.2019.01081)
Supplement: Supplementary file 1 [file Table_1.pdf]

frontiers

Supplementary Material

Supplementary Table 1. Overview of modules of the Workplace Mindfulness Training

Module	Goals	Methods
**Deepening day**	Getting to know Mindfulness	Mindfulness meditation<br>Walking meditation<br>Simple dialogs<br>Reflection and journaling
**Module 1: Attention and focus**	Understand and experience how multi-tasking negatively impacts attention and wellbeing.	Multi-tasking exercises<br>Pausing Meditation to cope with interruptions<br>Mindful e-Mailing
**Module 2: Emotions**	Understanding the neuroscience of emotions. Becoming curious about emotions and developing self-compassion.	Mindfulness practice<br>Dialogs on emotions<br>Body scan<br>Method of working with emotions
**Module 3: Happiness**	Learning how to cultivate happiness and how it impacts engagement and performance	Aimless wandering<br>Appreciative reflection
**Module 4: Time**	Understanding that we cannot manage time – but we can manage our attention. Learning about Flow.	Time sensation exercise<br>Reflection on time perception<br>One minute pause<br>Flow discussion
**Module 5: Review and transitions**	Deepening previous learning and strengthening practice through more practice	Mindfulness meditation<br>Rituals<br>Potentially Body Scan (20 min)
**Module 6: Communication**	Extending mindfulness in dealing with others.	Mindful listening<br>Unmindful listening<br>Deepening in mindful dialogs
**Module 7: Collaboration and trust**	Recognising that human relationships underpin all collaboration	Mindful dialogues<br>Perspective change/appreciative reflection<br>Mindful feedback<br>Mindful meetings<br>Compassion meditation
**Module 8: Self management and leadership**	Understanding the role mindfulness plays in leading our own life and in cultivating authenticity in leadership	Mindful walking<br>Space-awareness exercises<br>Reflection on decision making<br>Leadership exercise
**Final Day**	Reflecting the journey and commitment.	Walking through the module stations<br>Open Space – gathering of topics<br>World Café – Dialogue<br>Final presentation of topics
